# Supplementary material for: Changes of Volatile Flavor Compounds in Large Yellow Croaker (Larimichthys crocea) during Storage, as Evaluated by Headspace Gas Chromatography–Ion Mobility Spectrometry and Principal Component Analysis
Source: Foods. 2021 Nov 25;10(12):2917. doi: 10.3390/foods10122917 (PMC8701021; doi:10.3390/foods10122917)
Supplement: Supplementary file 1 [file foods-10-02917-s001.zip › foods-1453028-supplementary.pdf]

Supplementary Material

Table S1. Different sensors used to detect aromas by the electronic nose.

Name	Sensitivity Target	Name	Sensitivity Target
W1C	Aromatics	W5S	Nitrogen oxides
W3C	Ammonia and aromatic components	W6S	Hydride
W5C	Olefins and aromatic molecules	W1S	Methane
W1W	Sulfide	W2S	Ethanol and some aromatics
W2W	Organic sulfides	W3S	Alkanes and aliphatics
